# Drivers of Atherosclerotic Cardiovascular Disease in South Asians: Current Insights and Future Directions

**DOI:** 10.1016/j.jacasi.2025.10.024

**Published:** 2026-02-20

**Authors:** Aayush Patel, Stephen J. Nicholls, Derek P. Chew, Andrew K. Lin, Pradeep Natarajan, Adam J. Nelson, Nitesh Nerlekar

**Affiliations:** a Department of Cardiology, The Northern Hospital, Epping, Melbourne, Victoria, Australia; b Victorian Heart Institute, Monash University, Melbourne, Victoria, Australia; c Department of Cardiology, Victorian Heart Hospital, Melbourne, Victoria, Australia; d Baker Heart and Diabetes Institute, Melbourne, Victoria, Australia; e Cardiovascular Disease Initiative, Broad Institute of MIT and Harvard, Cambridge, Massachusetts, USA; f Center for Genomic Medicine and Cardiovascular Research Center, Department of Medicine, Massachusetts General Hospital, Boston, Massachusetts, USA; g Department of Medicine, Harvard Medical School, Boston, Massachusetts, USA; h Department of Cardiology, Royal Adelaide Hospital, Adelaide, Australia

**Keywords:** cardiovascular risk, review, South Asian

## Abstract

Atherosclerotic cardiovascular disease remains a leading cause of morbidity and mortality, with South Asians experiencing a disproportionately high burden. This excess risk is not fully explained by conventional risk factors, highlighting the need for a more nuanced understanding. However, progress has been hindered by a paucity of South Asian-specific research, limited validation studies, and under-representation in clinical trials. Emerging evidence points to a complex interplay of genetic predisposition, cardiometabolic dysfunction, lifestyle, and socioeconomic influences driving this heightened susceptibility. This review synthesizes recent advancements in genomics, lipid metabolism, biomarkers, imaging, and composite risk calculators, aiming to refine risk stratification in clinical practice. Additionally, we discuss key limitations in current research and explore future directions to bridge knowledge gaps. A deeper understanding of these factors is essential to developing targeted interventions and identifying novel therapeutic strategies to attenuate the elevated atherosclerotic cardiovascular disease risk.

## Highlights

• SA bear a disproportionately high burden of ASCVD and is projected to rise over the coming decades.
• Emerging research in genetics, cardiometabolic pathways, advanced imaging, and psychosocial determinants is improving our understanding of underlying drivers, refining risk stratification, and informing preventive strategies.
• SA-specific research and greater SA representation in clinical trials are essential to address existing knowledge gaps.

South Asian (SA) individuals represent people from Bangladesh, Bhutan, India, Maldives, Nepal, Pakistan, and Sri Lanka, together forming a quarter of the world’s population (1.91 billion).^1^ The SA diaspora is one of the fastest growing racial minorities in Western countries, forming 7.1%, 2.3%, and 1.7% of the total population of Canada, United Kingdom, and United States, respectively,^2^, ^3^, ^4^ and also represents a significant demographic in several key countries across Africa and the Caribbean. The burden of atherosclerotic cardiovascular disease (ASCVD) among SA individuals is substantial, with higher prevalence of subclinical atherosclerosis, earlier onset of symptomatic disease, and greater cardiovascular morbidity and mortality observed among resident and nonresident SA populations.^5^, ^6^, ^7^ There is a growing body of literature exploring its causal differences at genomic, metabolomic, and environmental levels to improve risk stratification and primary prevention using novel biomarkers, coronary imaging, and composite risk-stratification tools. This review summarizes the multifactorial drivers of increased risk from conventional risk factors as well as novel putative drivers, which may improve risk stratification, highlight opportunities for intervention, and reduce disease burden.

## Epidemiology

SAs experience clinical ASCVD almost 10 years earlier than other races (mean age 53 years)^8^ and demonstrate earlier development of subclinical disease than Caucasians.^9^ In 2019, ischemic heart disease (IHD) was the leading cause of mortality in South Asia, accounting for 105 deaths per 100,000.^10^ The residents of countries within South Asia also exhibit heterogeneous epidemiology, with the highest incidence observed in Bangladesh, and highest mortality seen in Pakistan.^11^ Similar variation was also observed in the SA diaspora of the UK BioBank study, with highest adjusted incidence rates observed among individuals of Bangladeshi and Pakistani origin, respectively.^12^ As a result of rapid population aging and expanding population size, by 2050, the crude cardiovascular mortality rate in South Asia is expected to rise by 85.3%, with ASCVD contributing to over one-half of these deaths.^13^

In the United States, SAs exhibit the highest prevalence of ASCVD (SA men 13.0% vs 8.5% Caucasian men) and the highest burden of mortality from IHD (proportionate mortality ratio 1.33).^6^ The risk of symptomatic ASCVD among SA diaspora in the United States and the United Kingdom is double that of Caucasians.^12^^,^^14^ This risk was only partially explained by differences in conventional risk factors.^12^ SAs also lead the incidence, prevalence, hospitalization, and mortality rates associated with ASCVD across several European countries.^15^

## Risk Factors and Pathophysiology

### Conventional risk factors

The burden of conventional risk factors for ASCVD, including hypertension, type 2 diabetes (T2D), dyslipidemia, and obesity, is higher among SAs than Caucasians across most higher income countries. Table 1 summarizes the observed prevalence of these risk factors among SAs in recent comparative studies. The frequency of hypertension varies between 18.4% and 37.4% among resident SAs with similar rates observed among SAs residing overseas.^16^ SAs exhibit a higher prevalence of body mass index (BMI) in the overweight category (25-29.9 kg/m^2^), but lower rates of true “obesity” (BMI ≥30 kg/m^2^) compared with Caucasians. SA men have a lower and SA women have a higher waist-to-hip ratio compared with Caucasians.^17^ At a given BMI or waist-to-hip ratio, SAs demonstrate a greater proportion of visceral adipose tissue than other races.^18^ Among resident SAs, the prevalence of abdominal obesity (waist circumference ≥90 cm [men], ≥80 cm [women]) is 39.5% (95% CI: 37.7%-41.4%).^19^

**Table 1:** Selected Cohort Studies Exploring Ethnic Differences in Conventional Risk Factors

	INTERHEART^46^ SA (n = 3,964) vs Other Ethnicities (n = 26,036)	UK BioBank^12^ SA (n = 8124) vs European (n = 449,349)		MASALA and MESA^164^ SA (n = 810) vs Non-Hispanic White (n = 2,622)	Pursnani et al^14^ (California, USA) SA (n = 5,149) vs White (n = 189,863)	
Hypertension, %	20.1 vs 32.3	41.8 vs 38.8		33 vs 33^a^	21.87 vs 31.44	
Type 2 diabetes, %	14.2 vs 12.9	19.5 vs 5.3		17 vs 5^a^	26.28 vs 16.38	
Dyslipidemia, %	**ApoB/ApoA1 ratio, %** 51.6 vs 40.3	20.9 vs 13.1^a^		43 vs 18^a^	25.19 vs 28.46^a^	
Total cholesterol, mg/dL		—		187 ± 37 vs 196 ± 35	192.17 ± 38.61 vs 201.33 ± 39.17	
LDL-C, mg/dL		130.9 ± 31.9 vs 139.3 ± 33.0		111 ± 32 vs 117 ± 30	114.88 ± 33.18 vs 120.38 ± 33.80	
HDL-C, mg/dL		49.0 ± 12.4 vs 56.5 ± 14.7		—	46.51 ± 11.41 vs 51.40 ± 14.77	
Triglycerides, mg/dL		148.7 (104.9-214.2) vs 131.2 (92.7-189.6)		—	155.03 ± 87.10 vs 148.83 ± 92.50	
Lp(a), nmol/L		31.2 (12.0-69.6) vs 18.5 (7.4-70.8)		—	—	
Body fat distribution	**Waist-hip ratio, %** 35.9 vs 40.5	**BMI, kg/m^2^**	27.2 ± 4.4 vs 27.3 ± 4.8	**Waist-hip ratio** 0.90 ± 0.07 vs 0.92 ± 0.09	**BMI 25-29.9 kg/m^2^, %**	36.43 vs 29.17
		**Waist-hip ratio**	0.90 ± 0.085 vs 0.87 ± 0.089		**BMI ≥30 kg/m^2^, %**	17.83 vs 33.98
Smoker, current or previous, %	50.0 vs 57.8	8.8 vs 10.4		—	20.55 vs 50.68	

Values are %, mean ± SD, or median (Q1-Q3). All *P* values <0.001.

HDL-C = high-density lipoprotein cholesterol; INTERHEART = a global study of risk factors for acute myocardial infarction; LDL-C = low-density lipoprotein cholesterol; Lp(a) = lipoprotein(a); MASALA = Mediators of Atherosclerosis in South Asians Living in America; MESA = Multiethnic Study of Atherosclerosis.

a Measured as currently taking pharmacotherapy. Comparative analyses using the new body mass index (BMI) recommendations for South Asians (SAs) (BMI 23-27.4 kg/m^2^ for overweight and BMI ≥27.5 kg/m^2^ for obese)^33^ have not been published.

Nonresident SAs have substantially lower rates of cigarette smoking compared with Caucasians (5.1% vs 18.5% current smoker, 8.1% vs 24.5% former smoker), among which SA women are even less likely to smoke than men (1.1% vs 8.7% current smoker, 1.4% vs 14.1% former smoker).^20^ Conversely, the prevalence of tobacco consumption is higher among resident SAs with highest rates observed among Maldivian (43.6%) and Bangladeshi men (39.8%). Among resident SA women, the prevalence of tobacco consumption ranges from 0.2% (Sri Lanka) and 8.3% (Nepal).^21^ Prospective studies have found that coronary heart disease-related mortality remains disproportionately high among SA men despite adjusting for conventional risk factors, other comorbidities, and socioeconomic status.^22^ Accordingly, SA ancestry is identified by the American College of Cardiology/American Heart Association (AHA) as a “risk-enhancing factor.”^23^

### Insulin resistance, type 2 diabetes, and gestational diabetes

Compared with Caucasians, nonresident SAs exhibit a higher age-adjusted prevalence of T2D (23% vs 6%)^24^ and are 5 years younger at diagnosis.^25^ The prevalence of T2D also varies within nonresident subpopulations (Indian 12.4%, Pakistani 16.9%, Bangladeshi 22.6%).^26^ Among resident SAs, the national prevalence of T2D is lower yet more disparate, ranging from 3.0% in Nepal to 8.3% in India and 9.6% in Bangladesh.^27^ T2D is significantly more common in urban sectors, with the urban-to-rural prevalence ratios of 1.4 (Pakistan), 1.9 (Sri Lanka), 2.4 (India), 3.5 (Bangladesh), and 5.8 (Nepal).^28^ This variation is influenced by health inequity and heterogeneity in screening guidelines across South Asia; yet, temporal trends demonstrate a surging prevalence of T2D driven by urbanization.^29^

Insulin resistance, closely linked to visceral adiposity and dysregulated adipokine secretion, is a key mechanistic driver of T2D, metabolic syndrome, and premature ASCVD in SAs.^30^ Genetic susceptibility, lower lean muscle mass, dysregulated lipid metabolism, and altered adipose distribution collectively impair β-cell function and insulin sensitivity.^31^ These intrinsic factors are further exacerbated by dietary patterns high in saturated fats, refined carbohydrates, and trans fats, alongside low intake of monounsaturated and polyunsaturated fats, sedentary lifestyle, and complex environmental and cultural factors, all contribute to the development of insulin resistance.^31^ SAs have higher plasma insulin levels, insulin-like growth factor-binding protein, and leptin levels, and lower adiponectin and resistin levels, which further contribute to insulin resistance and risk of T2D.^32^ SAs develop T2D at lower BMI thresholds than other races, with equivalent age- and sex-adjusted incidence observed at a BMI of 23.9 kg/m^2^ in SAs vs 30.0 kg/m^2^ in Caucasians.^33^ The MASALA (Mediators of Atherosclerosis in South Asians Living in America) study captured high rates (32%; 95% CI: 27.6%-35.9%) of progression from normoglycemia to prediabetes/T2D or prediabetes to T2D among nonresident SAs over 5 years.^27^ Similarly, an Indian study used Markov models to identify a rapid transition from impaired glucose tolerance (13.9%/year; 95% CI: 12.0%-15.9%) and isolated impaired fasting glucose (8.6%/year; 95% CI: 7.3%-9.8%) to established T2D over a 4-year follow-up.^34^

Gestational diabetes (GDM) is an established risk factor for T2D and is independently associated with future ASCVD risk. SA women have the highest prevalence of GDM, nearly double that of the general population, making it a significant emerging risk factor.^35^ GDM is associated with double the risk of coronary artery calcium (CAC) deposition,^36^ and increases the risk of cardiovascular disease among nonresident SA women.^37^^,^^38^ Local studies corroborate these conclusions, where resident SA women with GDM experience higher rates of T2D (22.2% vs 12.6%; *P =* 0.010, median 32 months)^39^ and a propensity toward adverse cardiovascular risk profile.^40^ Modifiable risk factors such as prepregnancy weight and low-quality diet only account for 37% of the population attributable risk of GDM.^41^ A T2D polygenic risk score has demonstrated a strong genetic susceptibility toward GDM in nonresident SA women,^42^ but these findings need to be validated in resident SA women. Newborns of SA mothers with GDM also have increased birth weight, total body fat, and reduced insulin sensitivity, which is postulated to contribute to adiposity and T2D in adulthood.^37^ Once diagnosed, a lack of culturally tailored education and lifestyle counselling, social stigma, pre-existing beliefs about antidiabetic drugs, and preference for traditional remedies may contribute to observed suboptimal glycemic control among SAs.^43^, ^44^, ^45^

### Obesity and fat distribution

Obesity and atherosclerosis share several mechanistic intermediates including dyslipidemia, T2D, and hypertension. Among SAs, the increased risk of ASCVD is more tightly linked to the quality and distribution of adipose tissue than absolute body weight. SAs are more likely to develop ASCVD at a lower BMI than other races.^46^ SA men and women exhibit 5% to 7% and 8% higher body fat percentage than Caucasians at a given BMI level, respectively.^47^ A meta-analysis identified that SA men but not women demonstrate higher subcutaneous fat (standardized mean difference [SMD] 0.34; 95% CI: 0.12-0.55) than Caucasians despite a lower mean BMI.^48^ More critically, SAs also exhibit higher age- and risk factor–adjusted visceral fat area than other non-Caucasian races (157 cm^2^ vs 135 cm^2^ Chinese vs 111 cm^2^ African American vs 141 cm^2^ Hispanic; all *P <* 0.05).^49^ Even from birth, SA infants demonstrate truncal obesity and atherogenic lipid profile, which are postulated to drive proinflammatory processes through the fetal programming hypothesis.^50^ Visceral fat volume is directly associated with the risk of ASCVD among SAs^51^ and partially accounts for higher rates of insulin resistance and metabolic syndrome seen in SAs.^50^^,^^52^^,^^53^ Longitudinal data from urban and rural India concur an early incidence of central obesity (19% at 28 years and 59% at 42 years), higher than expected incidence of comorbid hypertension and T2D (compared with other similar income countries), and higher rates of incident ASCVD; highlighting the need for early interventions.^54^

Conversely, subcutaneous adipose tissue is thought to be relatively benign. It is theorized that once subcutaneous storage capacity is exceeded, the “overflow” fat is stored in ectopic compartments including the abdomen and liver, which may be more pathogenic.^51^ Both SA men and women display greater liver fat than Caucasians (SMD 0.56; 95% CI: 0.14-0.98 and SMD 0.31; 95% CI: 0.14-0.48, respectively).^48^ The greater intrahepatic fat content suggests systemic disturbances in insulin signaling and lipid handling^55^ and possibly explains higher rates of ASCVD observed at lower BMI.^51^^,^^56^ As such, the World Health Organization recommends lower BMI cutoffs to prompt timely primary prevention interventions.^33^ U.S.-based studies highlight this burden of obesity with 77.6% of nonresident SAs reporting a BMI ≥23 kg/m^2^ (overweight) and nearly 40% (39.3% men, 36.8% women) having a BMI ≥27.5 kg/m^2^ (obese).^32^ Meanwhile, in India, for SAs the weighted prevalence of BMI ≥25kg/m^2^ is 28.6% (95% CI: 26.9%-30.3%).^19^

BMI, and to an extent waist circumference, does not adequately capture the ethnic heterogeneity in adipose tissue distribution and its contribution to cardiovascular risk.^55^ The updated definition of obesity as “a condition of excess adiposity that represents a risk to health” underscores the limitations of a BMI-based approach and the associated underdiagnosis and overdiagnosis related ramifications.^57^ Abdominal adipose tissue is a major source of proinflammatory adipokines such as interleukin-6, tumor necrosis factor-α, leptin, angiotensinogen, and resistin, which are all postulated to link insulin resistance to atherosclerosis.^58^ Adipose tissue also produces anti-inflammatory and antiatherosclerotic adipokines such as adiponectin. Microscopically, SA individuals demonstrate a propensity toward adipocyte hypertrophy (as opposed to hyperplasia as seen among Caucasians) leading to lower adiponectin levels at lower BMI and perpetuating the so-called “ethnic lipodystrophy.”^56^ Altered adipokine composition and abnormalities in the adiponectin-insulin sensitivity axis are thought to be implicated in the increased atherosclerotic risk seen in SAs.^59^^,^^60^ SAs have lower adipokine concentrations (Table 2), which is associated with insulin resistance, impaired fibrinolysis, and endothelial dysfunction.^61^

**Table 2:** A Summary of Key Differences in Biomarkers and Imaging Parameters Among SAs

	Observation	Relationship and statistical comparison			Risk Adjustment
Biomarkers					
High sensitivity C-reactive protein	↑CRP levels vs European, Chinese, and Canadian Aboriginals^66^	OR = 1.03 for a 0.1-mg/L increase in CRP; ***P =* 0.02** (n = 1,250)			Age, sex, CVRFs, atherosclerosis, anthropometry, race
PAI-1, ng/mL	↑PAI-1 activity vs Caucasians^165^	14.6 ± 2.2 vs 8.8 ± 2.4; ***P*** < **0.0005** in women, 21.3 ± 1.9 vs 12.2 ± 2.2; ***P*** < **0.0005** in men (n = 268)			Age, sex, BMI, cholesterol levels, fasting glucose
HDL-C, OR (95% CI)	↓HDL-C vs Caucasians^166^	3.90 (3.51-4.33) in women 2.94 (2.70-3.20) in men			Age, BMI, smoking status, CVRFs all ***P <* 0.001** (n = 169,430)
LDL-C, OR (95% CI)	↑LDL-C vs other races^166^	1.16 (1.04-1.30) in women 1.31 (1.21-1.43) in men			
TG, OR (95% CI)	↑TG vs other races^166^	2.10 (1.88-2.36) in women 2.62 (2.40-2.86) in men			
Apo A1	Lowest ApoA1 levels vs other Asians for a given HDL-C level^74^	***P* for heterogeneity** <**0.0001** (n = 9,699)			Age and sex
ApoB/ApoA1 ratio	↑ApoB/ApoA1 ratio vs Caucasians^167^	0.73 ± 0.04 vs 0.56 ± 0.03 in Women *± **P =*****0.0003** 0.85 ± 0.04 vs 0.66 ± 0.04 in Men *± **P =*****0.001**			—
Lp(a), nmol/L	↑Lp(a) vs NHW^168^	36 [19-79] vs 29 [13-72] **(*P =* 0.0085)** (n = 2,317)			—
	Independently associated with presence of CAD in Indians,^169^ OR (95% CI)	3.06 (1.24-7.55); ***P** <***0.001**			Age, gender, CVRFs
Mean adiponectin, μg/mL (95% CI)	↓Adiponectin vs Europeans^61^	9.35 (8.82–9.92) vs 12.96 (12.27–13.64) (n = 1,176) ***P*** < **0.001**			Age and waist circumference
Mean leptin, ng/mL (95% CI)	↑Adiponectin levels vs Europeans^61^	11.82 (10.72–13.04) vs 9.21 (8.38–10.12) (n = 1,176) ***P*** < **0.001**			Age and BMI
Imaging parameters					
EAT volume, cm^3^	↑EAT vs Caucasians^63^	103.2 ± 41.7 vs 85.8 ± 39.4; ***P =* 0.006** (n = 100)			—
Intrahepatic fat attenuation, Hounsfield Units	↑Intrahepatic fat vs Caucasians, Black, Latino and Chinese^55^	55 ± 11 vs 61 ± 12, 63 ± 12, 59 ± 14, 62 ± 12; ***P*** < **0.001** (n = 7,617)			—
CAC score, RR vs SA (95% CI)	↑CAC scores vs Black and Latino ∼ vs White and Chinese^100^ (n = 7,617)	White	0.99 (0.87–1.12)	*P* = 0.83	Sex, age, education, smoking, BMI, CVRFs, fasting glucose, insulin, and cholesterol levels
		Black	0.73 (0.65–0.83)	***P <* 0.001**	
		Latino	0.81 (0.71–0.92)	***P* = 0.001**	
		Chinese	0.95 (0.83–1.10)	*p* = 0.52	
CAC progression, fold change (95%CI)	∼ CAC progression vs White, but ↑ vs Black, Latino and Chinese^101^ (n = 6,382)	White	1.06 (0.82–1.36)	*p* = 0.65	Age, sex, smoking, CVRFs, body composition, lifestyle behaviors, medications
		Black	0.45 (0.34–0.58)	***P <* 0.001**	
		Latino	0.61 (0.46–0.79)	***P <* 0.001**	
		Chinese	0.65 (0.48–0.88)	***P* = 0.005**	
CAC volume and density, beta coefficient (95% CI)	↓ CAC Volume and ↑ CAC Density vs NHW^170^ (n = 3,433)	CAC volume	−0.46 (−0.62 to −0.29)	***P <* 0.05**	Age, sex, education, cholesterol-lowering therapy, antihypertensive use, CVRFs, smoking, (and reciprocally CAC density and volume)
		CAC density	0.14 (0.11-0.18)	***P <* 0.05**	
Number of calcified vessels, beta coefficient (95% CI)	↑number of vessels with CAC vs NHW^170^	0.18 (0.07-0.28); ***P*** < **0.05** (n = 3,433)			
LAD aggregate plaque volume, %	↑APV vs Caucasians and Southeast Asians^63^	44.5 ± 8.4 vs 39.5 ± 6.4, 37.5 ± 6.5, ***P* = 0.000002** (n = 150)			Age, gender, BMI, CVRFs, Statin use, Duke prognostic index, Segment stenosis score

Values are mean ± SD or median [Q1-Q3] unless otherwise indicated. Significant *P* values ≤ 0.05 are indicated in **bold**.

Apo = apolipoprotein; BMI = body mass index; CAC = coronary artery calcium; CVRF = cardiovascular risk factor (conventional); EAT = epicardial adipose tissue; Lp(a) = lipoprotein (a); NHW = non-Hispanic Whites; PAI-1 = plasminogen activator inhibitor type 1; SA = South Asian; TG = triglycerides.

Epicardial fat is strongly implicated with atherogenesis.^62^ SAs exhibit a greater volume of epicardial adipose tissue (EAT) than Caucasians.^63^ Pericardial fat volume, a composite of paracardial and epicardial fat, is weakly associated with CAC severity in SAs but does not completely explain the higher CAC burden.^55^ EAT may be a marker of increased metabolic and/or inflammatory perturbation, although it remains unclear whether reducing EAT can reliably attenuate the incidence or progression of ASCVD.

### Biomarkers

#### Inflammation

High sensitivity c-reactive protein (hsCRP) remains a predictor of ASCVD risk despite adjustment for conventional risk factors in addition to race and body habitus (Table 2).^64^ SAs demonstrate higher age- and sex-adjusted hsCRP levels than Chinese and European individuals and may reflect a higher prevalence of abdominal obesity in SAs.^65^^,^^66^ Accordingly, hsCRP *>*2 mg/L is identified as a risk modifier by the Lipid Association of India (LAI) guidelines,^67^ and it may help guide decision-making regarding the need for lipid-lowering therapy in moderate risk SAs.^41^

#### Cholesterol metabolism

Dyslipidemia is highly prevalent among SAs with a weighted prevalence of 81.2% (95% CI: 77.9%-84.5%) among resident SAs.^19^ It is classically characterized by elevated triglycerides, low levels of high-density lipoprotein cholesterol (HDL-C),^50^ and relatively lower levels of low-density lipoprotein cholesterol (LDL-C) compared with Caucasians (Table 1). This phenotype is also seen in resident SAs: in an Indian epidemiological study, the prevalence of hypertriglyceridemia and low HDL-C were 29.5% and 72.3%, respectively, with only 11.8% exhibiting elevated LDL-C levels.^68^ LDL-C and total cholesterol underestimate cardiovascular risk, as nearly one-quarter of nonresident SAs with coronary artery disease (CAD) have LDL-C <100 mg/dL and total cholesterol <150 mg/dL.^69^ SAs often exhibit small and dense LDL-C particles, which may be higher in particle number despite “normal” LDL-C concentrations. Elevated triglyceride levels, low HDL-C, and small dense LDL-C particles are postulated to be driven by insulin resistance and are considered atherogenic.^8^ Among those with existing CAD, small dense LDL-C is also the most important predictor of residual risk of future cardiovascular events despite statin use and normal LDL-C concentrations, but this is yet to be validated in SAs.^70^ Triglycerides and HDL-C are inexorably linked through lipoprotein metabolism whereby low levels of HDL-C frequently accompany elevated levels of atherogenic triglyceride rich lipoproteins (TRLs). SAs exhibit 30% higher activity of cholesteryl ester transfer protein, which promotes the transfer of cholesteryl esters from HDL-C to atherogenic lipoproteins resulting in a similar dyslipidemic profile.^71^ Even when present in normal concentrations, HDL particles among SAs appear to be smaller and more likely to be dysfunctional thereby attenuating any protection that may normally be afforded.^72^ Apolipoprotein A1 (ApoA1) is involved in the production of HDL-C and exhibits anti-inflammatory, antiatherogenic, and antithrombotic properties.^73^ ApoA1 levels demonstrate a linear association with HDL-C among all races, but SAs have the lowest ApoA1 levels at a given HDL-C level than other races (Table 2). In SAs, higher ApoA1 levels correlate more strongly with reduced acute myocardial infarction (AMI) risk than HDL-C.^74^ Apolipoprotein B (ApoB) is found in most atherogenic lipid particles including LDL, triglyceride-rich lipoproteins, and lipoprotein(a) [Lp(a)], providing a holistic assessment of lipid-related ASCVD risk.^75^ An elevated ApoB/ApoA1 ratio is a powerful risk factor for ASCVD, with SAs demonstrating higher ApoB/ApoA1 ratios than Caucasians despite a lower total cholesterol level.^74^ Despite the ApoB/ApoA1 ratio demonstrating the strongest association with the risk of AMI in SAs, widespread adoption has been limited because of the established LDL-C paradigm, the paucity of affordable and standardized assays, and the absence of therapeutic treatment targets. However, recent preventative health literature endorses ApoB testing in comprehensive dyslipidemia assessment and therapeutic monitoring.^76^

#### Lipoprotein(a)

Lp(a) is an apo-B containing lipoprotein that is associated with the development of both ASCVD and calcific aortic stenosis.^22^ Mendelian randomization studies strongly suggest that Lp(a) causally influences these conditions.^77^ The population-attributable risk of Lp(a) for AMI is 9.5% in SAs, double that of Caucasian populations, and similar to that of conventional risk factors like diabetes.^78^ This may be driven by a higher ancestral presence of elevated Lp(a), with nearly one-quarter of resident and nonresident SAs demonstrating Lp(a) ≥50 mg/dL (125 nmol/L).^78^^,^^79^ An elevated Lp(a) in conjunction with a positive family history also confers an independent and additive association in predicting cardiovascular risk.^80^^,^^81^ Lp(a) levels remain fairly static throughout life,^82^ making it an attractive biomarker for lifetime ASCVD risk estimation and guide primary prevention interventions. Current guidelines recommend a once-in-a-lifetime measurement of Lp(a) in high-risk individuals with a positive family history.^67^^,^^83^ The LAI guidelines additionally recommend screening for Lp(a) at or before age 18 years to better assess the risk of premature ASCVD. An Lp(a) level ≥50 mg/dL is considered a high-risk feature, whereas levels between 20 to 49 mg/dL are viewed as risk modifiers that may warrant upstaging individuals from low or moderate risk categories.^67^ Proprotein convertase subtilisin/kexin type 9 inhibitors are capable of lowering Lp(a) levels by 20% to 25%^84^; however, there are currently no approved therapies to specifically lower Lp(a) levels. Clinical trials of multiple agents capable of lowering levels by >90% from baseline are ongoing and will determine whether Lp(a) moves forward in the clinic from risk marker to therapeutic target.^85^

## Current Approaches To Risk Assessment

Current recommendations for risk assessment include assessment and management of key clinical and biochemical risk factors highlighted in the previous text. SA-specific guidelines or treatment targets do not exist. However, emerging literature recommends a target BMI ≤23 kg/m^2^ and earlier screening for dyslipidemia in high-risk patients.^32^ Risk estimating equations and CAC scoring can help further stratify the ASCVD risk.

### Risk estimating equations

Several equations have been developed to estimate the risk of ASCVD. The American College of Cardiology/AHA Pooled Cohort Equations (PCE) and the modified Framingham Risk Score (FRS) are commonly used in the United States, whereas the SCORE tool is utilized in the United Kingdom and Europe. SAs were not well represented in the prospective cohort data sets used in the training of these equations, and thus accuracy among SAs is less optimal.^12^^,^^86^^,^^87^ Furthermore, they do not account for duration of residency outside of SA, generational status, or mixed-racial ancestry—all factors that may modulate ASCVD risk.^59^

Risk estimation for resident SAs is even more challenging because there are no calculators prospectively validated in the native population, while retrospective data are rather conflicting. In 2 separate retrospective studies, Bansal et al^88^^,^^89^ found the 3^rd^ Joint British Societies (JBS3) calculator to be the most accurate at identifying Indians with AMI as high-risk^88^ and observed the best correlation with CAC score.^89^ Conversely, Garg et al^90^ found the FRS-CVD score to be most accurate in identifying SA patients with AMI as “high-risk,” with intermediate performance observed by QRISK2 and JBS3. British risk scores like JBS3 and QRISK3 are thought to be better suited for risk prediction because of better SA representation in the derivation cohort (2.3% for JBS3, 4.1% for QRISK3).^91^ The updated QRISK3 also accounts for heterogeneity within SAs with different risk multipliers recommended for men and women from India, Pakistan, and Bangladesh.^92^ As such, the LAI endorses the use of QRISK3 score and further highlights the need for a large-scale prospective database to develop a dedicated risk calculator fit for population-specific use.^67^ Meanwhile, calculators with better SA representation—such as ETHRISK (United Kingdom, 46% SAs), NORRISK 2-SADia (Norway, 12%)—although endorsed by their respective national guidelines, are not explicitly recommended over other equations for ASCVD risk profiling in resident SAs.^32^ A machine learning-based Sri Lankan Cardiovascular Disease score has recently been developed using an all Sri Lankan cohort (n = 2,596), demonstrating superior 10-year predictive accuracy compared with the World Health Organization (WHO) risk charts.^93^ Although its performance is yet to be validated in larger cohorts and benchmarked against more widely used risk calculators, such novel approaches may help address the current limitations in ASCVD risk estimation specific to resident SAs.

Miscalibration of ASCVD risk can lead to adverse implications. Many calculators such as the modified FRS and the SCORE tool utilized crude multipliers based on race or country of origin to address the underestimation of ASCVD risk observed among SAs in retrospective studies.^94^^,^^95^ Conversely, the PCE overestimates risk in borderline- and intermediate-risk SAs, because 30% and 54%, respectively, of these patients were found to have a CAC score of zero, corresponding to low ASCVD risk, and possibly at risk of overtreatment.^96^ The LAI guidelines emphasize the calculation of lifetime risk using QRISK3 to better stratify SAs with low 10-year risk and implement primary prevention interventions early.^67^ The predictive accuracy of risk calculators in identifying borderline- or intermediate-risk resident SAs remains largely unexplored. Yet, this group may represent a critical cohort for targeted primary prevention, with the greatest potential to attenuate their ASCVD risk.

The AHA’s novel PREVENT equations estimate long-term cardiometabolic risk and intentionally exclude race and ethnicity as a variable.^97^ Although Asians only represented <3% of the derivation and validation cohorts, the inclusion of zip code–based social deprivation index is thought to better capture the socioeconomic factors that influence ASCVD risk, improving risk modelling in historically marginalized communities such as SAs residing in the United States. However, these equations have yet to be validated for SAs residing outside of the United States. PREVENT also omits positive family history as a parameter and may fail to capture the inherited genetic contribution in some high-risk SAs. External validation studies with prospective SA cohorts are needed to better assess its predictive accuracy.

### Imaging

#### Coronary Artery Calcium

CAC testing is an established screening method for detecting subclinical atherosclerosis and correlates with future ASCVD risk among all races, including resident and nonresident SAs.^75^^,^^98^^,^^99^ After adjusting for conventional risk factors, the CAC burden in SA men and Caucasian men is similar but higher than other races.^100^ CAC density and CAC volume also correlate with estimated ASCVD risk using risk calculators despite adjusting for conventional risk factors. SA men have similar CAC progression to European men but greater CAC progression than men of other races after adjusting for conventional risk factors. SA women exhibit similar CAC incidence and CAC progression rates compared to women of other races.^101^ SAs exhibit significantly lower CAC volume but higher CAC density than Caucasians.^102^ Higher CAC density is thought to be associated with stable plaque, is less likely to rupture, and may be influenced by the procalcific effects of statin use.^32^^,^^102^

Intermediate-risk SAs (as per PCE) are more likely to have CAC score of 0 than Caucasians (OR: 1.72; 95% CI: 1.00-2.99), corresponding to very low future ASCVD risk.^96^ As such, CAC scoring may help reclassify SAs who are at the greatest risk of overestimation using risk calculators and serve as a guide for initiating or deferring preventative interventions. SAs with a positive family history are more likely to have CAC >300, equating to very high risk, than individuals of other races (OR: 2.82; 95% CI: 1.6-4.93). Current guidelines recommend CAC scoring for SA adults over 40 years of age who are at higher risk than estimated by traditional risk stratification tools including those with significant family history of premature ASCVD, those with predisposing comorbidities such as prediabetes and metabolic syndrome, or those with 2 or more risk-enhancing factors.^98^^,^^103^ However, SAs remain at a high risk of premature acute coronary syndrome (ACS) with one-half of the episodes occurring before the age of 50 years and 25% before the age of 40 years.^8^ SA-specific CAC percentiles and screening guidelines, particularly in those aged <40 years, are warranted for enhanced risk stratification. Although clinically useful, CAC testing does not necessarily capture the excess risk among SAs, especially among young SAs who demonstrate a high prevalence of noncalcified, low-attenuation plaque that is associated with AMI among other races.^76^

#### Plaque characteristics

On coronary computed tomography angiography (CTA), SAs have greater volume and more advanced plaque phenotypes compared with other races and ethnicities.^98^ In a small coronary CTA study, the volume of atherosclerotic plaque among SAs was higher despite adjustment for age, sex, and BMI.^63^ Asymptomatic SAs with T2D were more likely to have >50% stenosis of the left anterior descending coronary artery than Caucasians with T2D. In a cross-sectional study using high-resolution intracoronary imaging, plaque in SAs had more prevalent vulnerable characteristics including greater lipid arc and higher incidence of thin fibrous caps. These SAs were more likely to present with ST-segment elevation myocardial infarction at a younger age, with plaque erosion being the predominant pathology.^104^ The long-term clinical and prognostic significance of these parameters in predicting cardiac risk is unknown and remains an area of interest for future research.^98^

Management of any SA individual with subclinical ASCVD on coronary CTA or CAC scoring requires intensive risk factor modification and preventative cardiometabolic. Although there is evidence of CAC-guided intervention to slow plaque progression,^105^ its effects on long-term risk of symptomatic CAD and major adverse cardiac events are uncertain among SAs. Surrogates of subclinical atherosclerosis imaging such as CAC warrant further exploration given that it is a truly personalized marker capable of identifying early disease formation.

## Emerging Multi-Omics Approaches

Current recommendations fail to completely encapsulate the elevated risk observed in SAs. To better elucidate the underlying drivers of atherosclerosis, academic discourse is shifting toward a multi-omics approach integrating genomics, proteomics, metabolomics, and socioeconomics (Central Illustration). This comprehensive approach holds promise for the identification of novel biomarkers, therapeutic targets, enhanced risk stratification, and personalized intervention strategies.^76^

**Central Illustration:**
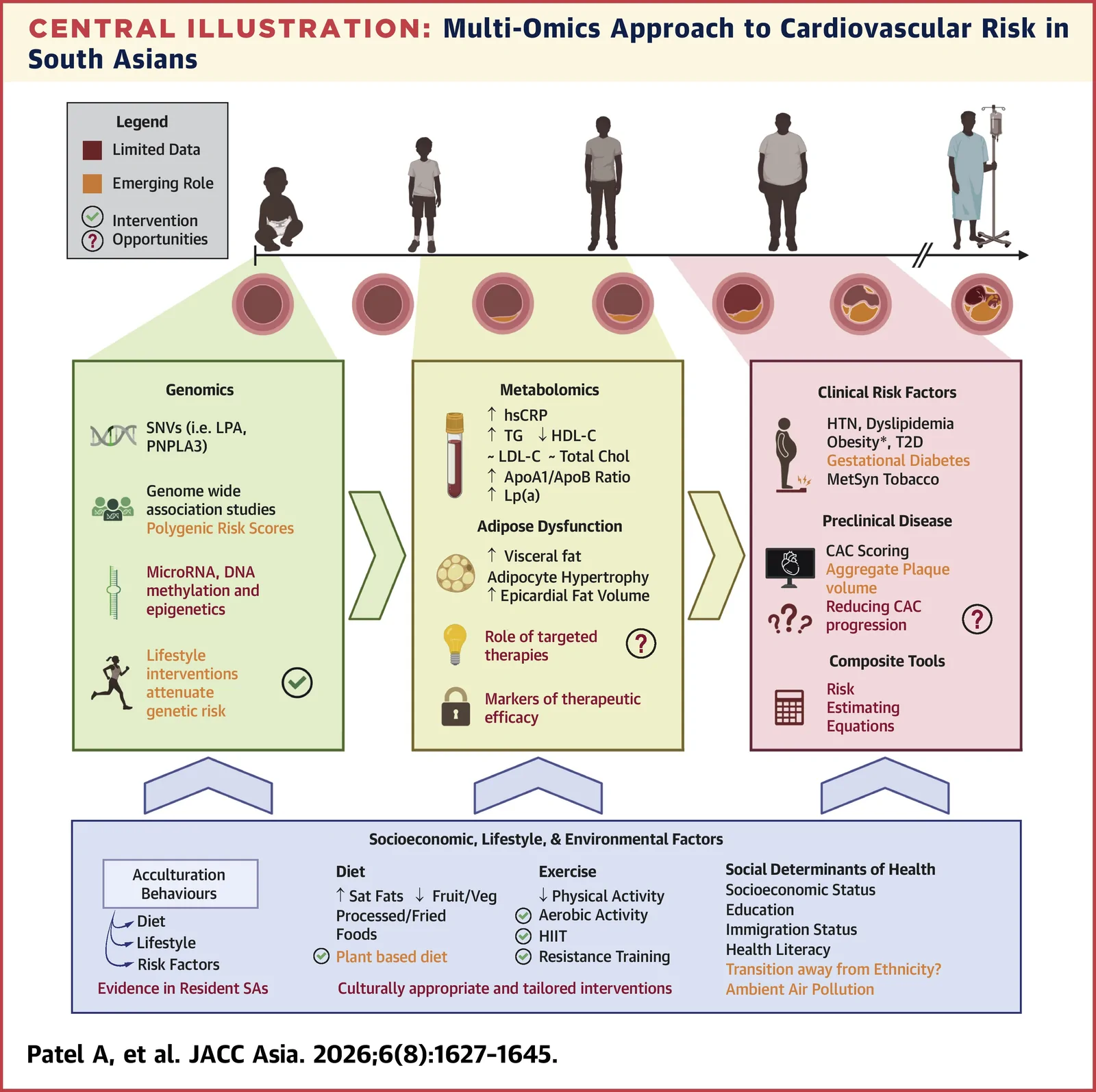
Multi-Omics Approach to Cardiovascular Risk in South Asians Conceptual framework illustrating the multi-omics interplay in the development and progression of atherosclerotic cardiovascular disease in South Asian (SA) populations. Genetic and metabolic susceptibility converge with lifestyle, socioeconomic, and cultural factors to shape preclinical disease and clinical outcomes. Icons denote emerging roles (yellow), areas with limited data (red), and opportunities for intervention, highlighting priorities for improving primary prevention and guiding future research in SAs. ApoA1 = apolipoprotein A1; ApoB = apolipoprotein B; CAC = coronary artery calcium; Chol = cholesterol; HDL-C = high density lipoprotein cholesterol; HIIT = high intensity interval training; hsCRP = high sensitivity C-reactive protein; HTN = hypertension; LDL-C = low density lipoprotein cholesterol; Lp(a) = lipoprotein (a); MetSyn = metabolic syndrome; Sat Fats = saturated fats; SNVs = single nucleotide variations; T2D = type 2 diabetes; TG = triglycerides.

### Genomics

Genetic differences may elucidate some of the racial differences in ASCVD unexplained by conventional risk factors. Genome-wide association studies (GWAS) and sequencing studies have begun to identify SA-specific genetic variants associated with cardiometabolic risk.

Variation in the *LPA* gene is one of the commonest genetic risk factors for premature ASCVD and codes for the Lp(a) particle. Several single nucleotide variations (SNVs) have been identified in the *LPA* locus, conferring up to a 1.58-fold increased risk of developing CAD.^22^^,^^106^ Up to 70% of the *LPA* coding sequence is in the hyper-variable kringle IV type 2 (KIV-2) region consisting of variable tandem repeats. The number of KIV-2 repeats inversely correlate with the expression of Lp(a), the plasma Lp(a) levels, and the subsequent risk of ASCVD.^22^ However, *LPA* SNVs and KIV-2 copy number together explain a small proportion of Lp(a) variation observed in SAs (21% vs 36% in Caucasians and 27% in Chinese).^107^

The Pakistan PROMIS (Risk of Myocardial Infarction Study) sequenced the protein-coding regions among 10,503 individuals and identified homozygous loss-of-function alterations in the coding region of *APOC3*, a gene known to harbor cardioprotective variants.^108^ A meta-analysis of 4 GWAS (n = 15,420; 6,996 SAs) identified 5 novel loci associated with ASCVD risk in SAs: *LIPA* (10q23), *PDGFD* (11q22), *ADAMTS7-MORF4L1* (15q25), a gene-rich region on 7q22, and *KIAA1462* (10p11).^109^ Although this analysis significantly expanded the number of confirmed CAD susceptibility loci, it found no major differences in effect size or allele frequency between SAs and Europeans—highlighting the limitation that current GWAS arrays may miss population-specific variants relevant to SAs. A GWAS of T2D in SAs (5,561 cases, 14,458 control subjects) identified 20 SNVs associated with T2D in an all-SA replication sample of 13,170 cases and 25,398 control subjects.^110^ Six of these variant loci (*GRB14, ST6GAL1, VPS26A, HMG20A, AP3S2*, and *HNF4A*) were newly associated with T2D with SNVs at *GRB14* additionally associated with insulin sensitivity and SNVs at *ST6GAL1* and *HNF4A* associated with pancreatic beta cell function—emphasizing the need for dedicated SA studies to identify novel genetic variants that incrementally contribute to ASCVD risk.

Monogenic variants such as familial hypercholesterolemia (FH) are equally common among SAs (estimated prevalence of variant *FH* gene 1:226 vs 1:288 in Europeans).^111^ However, most of the genetic risk is thought to be a cumulative effect of many commonly inherited DNA variants, across the whole genome.^112^ This theory has led to the development of various polygenic risk scores (PRS) to better assess genetic risk. Unfortunately, derivation cohorts have been of predominantly European ancestry, and such scores have an attenuated efficacy when applied to other races.^113^^,^^114^ Unfortunately, SA-specific PRS studies are limited, often validated in small cohorts, and consequently underpowered. Wang and colleagues derived a new PRS model utilizing the summary statistics from a prior GWAS, validated in 7,244 SA UK BioBank participants and tested this framework in predicting the genetic risk of ASCVD in an Indian cohort (1,800 with CAD, 1,163 control subjects). They identified a 3.22-fold increase in disease risk between the highest and lower risk quintiles of SA population (OR/SD: 1.58; 95% CI: 1.42-1.76) with good performance (area under the receiver-operator curve = 0.80).^113^ Independent of conventional risk factors, the SAs in the top 5 percentile of genetic risk were 4.16 times more likely to develop CAD than the median.^113^ The addition of PRS is also shown to improve the predictive performance of risk estimating equations across all races, including SAs (net reclassification index, 6.7%; 95% CI: 3.1-14.4).^115^ Ancestry-specific PRS may further improve predictive accuracy, allowing elevated risk SAs to be identified early and be offered intensive primary prevention strategies.

Genotypic arrays as well as low-coverage whole genome sequencing have become increasingly inexpensive and more readily available.^112^ Risk models can also be improved with additional participant data sets and fine-tuned with larger-scale validation studies.^113^ Polygenic risk scoring remains an emerging field, with the potential to evolve into a valuable population-level screening tool for enhanced ASCVD risk stratification.

#### Epigenetics

Epigenetic alterations contribute to the intergenerational inheritance of genetic ASCVD risk. The 3 primary mechanisms that facilitate epimutation of phenotypes include DNA methylation, core histone protein modification, and alteration of microRNA (miR) expression.^22^ Epigenetic association studies exploring ASCVD and cardiometabolic risk factors is an emerging field with limited SA-specific data. A nested case-control study of the LOLIPOP (London Life Sciences Prospective Population) cohort (13,535 Indian Asians and 7,066 Europeans) has identified DNA methylation at 5 genetic loci (*ABCG1, PHOSPHO1, SOCS3, SREBF1*, and *TXNIP*) to be predictive of future T2D risk over a mean 8.5-year follow-up period. The differences in DNA methylation accounted for a significant amount of previously unexplained increased risk of T2D relative to Europeans.^116^ A small Pakistani cross-sectional study (n = 203) utilized methylation-specific PCR to identify DNA methylation of 5 key genes (*Chemerin, IGF2, POMC, PCSK1* [*P <* 0.001], and *FNDC* [*P <* 0.05]) associated with obesity and metabolic syndrome.^117^ DNA methylation may be a future epigenetic marker in identifying SAs who may benefit from early interventions, but larger-scale validation studies are needed in the first instance.

A study of 128 nonresident Asian Indians used flow cytometry-based assays to identify 6 miRs to be negatively associated with glycemic progression on oral glucose tolerance tests at 2.5 years.^118^ Another nested case-control study of 44 Indian men identified altered expression of 15 miRs on flow cytometry in the dyslipidemia group, of which 3 miRs are believed to target the transcription of genes involved in lipid metabolism.^119^ Validation of the epigenetic mechanism of such miRs in the SA cohort is an area of promise as miR can be found in peripheral blood, used as a biomarker to measure ASCVD risk in clinical practice, and serve as a therapeutic intervention target among asymptomatic SAs.

### Socioeconomics and cultural factors

Social determinants of health such as household income, level of education, diet and immigration status affect health care access and quality. Country of residence may play a significant role in the development of ASCVD. Nonresident SAs exhibit a degree of acculturation toward a Western diet and lifestyle with propensity toward higher health literacy, increased access to care and higher participation in preventative health care. Two of the most important studies investigating ASCVD in SAs (MASALA and the UK BioBank Study) capture the nonresident SAs in the United States and United Kingdom, respectively.^12^^,^^120^

Acculturation is the social paradigm where immigrant populations adapt to a new cultural environment including behaviors that influence health such as diet, physical activity, and substance use. The most common acculturation behaviors seen in SA populations are as follows: 1) assimilation, where the customs of the new environment are embraced often at the detriment of native customs; 2) separation, where prior customs are strongly maintained and customs of the new environment are rejected; and 3) integration, where a combination of native customs are maintained while adopting aspects of the new culture.^8^ The MASALA study found a 23%, 23%, and 54% distribution of acculturation strategies among SAs residing in the United States, respectively. Interestingly, SA women (but not men) in the integration and assimilation strategies were found to have a more favorable cardiometabolic risk profile.^121^ Separation behaviors are thought to be exhibited by older generations, which may be contributed to by language barriers and risks suboptimal access to health care and worse management of ASCVD risk factors.^8^ In SAs, risk factors for ASCVD, diet and exercise patterns vary by degree of acculturation, typically measured by birthplace and duration of residency.^6^ Greater acculturation to the United States has been associated with increased ASCVD risk and poorer cardiovascular health metrices. However, recent data from the United Kingdom show substantial attenuation in excess cardiovascular mortality previously seen in SAs with T2D, with mortality rates now lower than Caucasians in some studies.^122^ This conflicting trend is postulated to be caused by earlier diagnosis of T2D among SAs, leading to enhanced opportunistic screening and management of other cardiovascular risk factors and possibly earlier commencement of pharmacotherapies for hypertension and dyslipidemia. Moreover, a higher proportion of second-generation migrants with better health literacy, health equity, and lower smoking rates alongside disproportionately rising rates of obesity among Caucasians may also contribute toward this observation.

An overwhelming majority of SAs live within their country of origin; yet, large-scale data investigating ASCVD among resident SAs are limited. Individuals who emigrate to Western countries may already belong to a higher socioeconomic status, with better health literacy and improved access to health care. Their financial and socioeconomic status may improve further following emigration, thus influencing their diet and lifestyle patterns; hence, lifestyle recommendations for nonresident SAs do not universally apply to natively residing SAs, and lifestyle interventions should be tailored and validated for their efficacy in each geo-cultural setting, whether it be regional Sri Lanka or metropolitan India. Conversely, environmental factors like pollution and stress may be less relevant to nonresident SAs.

Indian epidemiological studies have identified a significant disparity in the prevalence of common cardiometabolic disorders between urban and rural cohorts. Individuals residing in urban areas are more likely to have diabetes (31.8% vs 24.1%), hypertension (40.7% vs 33.0%), BMI ≥25 kg/m^2^ (39.6% vs 23.1%), and abdominal obesity (51.6% vs 33.5%) than rurally residing cohorts (all *P <* 0.0001; n = 113,043).^19^ The observed variation in the dyslipidemia profile (total cholesterol, triglycerides, HDL-C, LDL-C) was substantially smaller between urban and rural cohorts. Adverse dietary and lifestyle patterns in the higher socioeconomic groups as well as environmental exposures are thought to explain this trend.^123^

### Lifestyle factors

#### Diet

A typical SA diet is high in refined carbohydrates, saturated fats (ie, ghee, coconut oil, and palm oil), and trans fats (ie, repeated use of oil in deep frying, partially hydrogenated vegetable oils), and low in fruits and vegetables. Among resident SAs, “Prudent” dietary pattern with controlled consumption of fat- and cholesterol-containing foods is associated with lower rates of T2D, and a higher dietary diversity is associated with lower rates of T2D and hypertension.^124^ SAs who follow a vegetarian diet have been shown to exhibit lower rates of insulin resistance, total cholesterol, LDL-C, triglycerides, and systolic and diastolic blood pressure, and lower fasting glucose levels.^125^^,^^126^ High fruit and vegetable consumption in SAs is associated with a lower risk of cardiometabolic risk factors and ASCVD risk^127^^,^^128^; however, despite a large proportion of SAs being vegetarian, the overall fruit and vegetable consumption among SAs is low.^129^ Vegetarian SA diets are higher in saturated fats, processed foods, and total caloric content, making them more atherogenic than other plant-based diets. Nonvegetarian SAs also consume low amounts of fish compared with other Europeans, which may explain the attenuated reduction in cardiometabolic risk factors observed.^126^ Acculturation also influences dietary patterns among the nonresident SA population. Cross-sectional investigation of the MASALA study identified that an assimilation strategy with weaker traditional cultural beliefs was associated with more of the animal protein dietary pattern, which is associated with higher BMI and LDL-C. SAs with stronger traditional cultural beliefs were more likely to consume a diet consisting of fried snacks, sweets, and high-fat dairy, which is linked to lower HDL-C and greater insulin resistance.^130^

#### Exercise

Sedentary lifestyle is linked with higher BMI, waist circumference, systolic blood pressure, and suboptimal glycemic control.^131^^,^^132^ SAs are generally less physically active than individuals from other racial groups.^131^ SA women are even less physically active than men.^133^ A cross-sectional study identified a 28% lower ASCVD rate among native SAs who performed the WHO recommended 150 minutes of moderate-intensity or 75 minutes of high-intensity exercise.^134^ Moreover, some physical activity was associated with 12% lower rates of ASCVD compared with physical inactivity. This highlights the importance of improving awareness of the interplay of physical activity and ASCVD prevention.^6^^,^^125^ Perceived barriers to engagement with physical activity include lack of knowledge and understanding as well as pre-existing cultural beliefs and perceptions, highlighting a role for culturally tailored counselling and exercise programmes.^135^

### Environmental factors

Exposure to chronic air pollution is an established risk factor for ASCVD and other cardiometabolic comorbidities, although the underlying pathophysiological mechanisms remain elusive. The rapid urbanization and industrial expansion of South Asian countries has led to unprecedented rise in biomass fuel use and elevated fine particulate matter levels (ie, PM2.5) and 60% of resident SAs are exposed to hazardous levels of pollution.^136^ In total, 37 of the world’s top 40 most polluted cities are in South Asia, and 20% of deaths from IHD in SA can be attributed to ambient air pollution (vs 15% among the rest of the world).^137^ The role of longitudinal ambient air pollution exposure in ASCVD incidence risk among SAs and whether it differs from other populations is not known. Studies originating from developed countries cannot be generalized to South Asia caused by the observed differences in pollutants, their sources, and concentrations. Cross-sectional data predominantly originating from India have demonstrated a positive association between air pollution (composite particulate matter and specific pollutants) and biomarkers of oxidative stress and inflammation as well as traditional cardiovascular risk factors such as hypertension.^138^^,^^139^ However, longitudinal data to facilitate risk estimation for SAs are lacking. Emerging literature points toward other forms of pollution, such as water, soil, noise, and light, as potential drivers of ASCVD.^140^ Concerted academic and public health efforts are required at local and international levels to better understand, predict and attenuate the downstream effects of pollution on ASCVD among SAs.

## Opportunities for Intervention Specific to SAs

Diet, lifestyle, and physical activity-based interventions are cornerstones of ASCVD prevention regardless of race. A favorable lifestyle consisting of at least 3 of the following—no current smoking, no obesity, regular physical activity, and a healthy diet—is demonstrated to dramatically reduce the risk of coronary events, even in those deemed at a genetically high risk by PRS (46% lower relative risk; HR: 0.54; 95% CI: 0.47-0.63).^141^ Therefore, even high-risk SAs with a seemingly nonmodifiable genetic predisposition to ASCVD can benefit from preventative lifestyle interventions.

Transition to a plant-based diet improves glycosylated hemoglobin, visceral fat, pericardial fat, metabolic dysfunction-associated steatotic liver disease, and cardiometabolic profile among SAs at 5 years.^125^ Adherence to a high Dietary Approaches to Stop Hypertension (DASH) diet is associated with slower CAC progression in SAs; however, larger prospective studies are still required for validation.^142^ A novel South Asian Mediterranean-style (SAM) diet has been shown to lower glycosylated hemoglobin, pericardial fat volume, risk of obesity, fatty liver, and long-term incidence of T2D.^143^ Current SA guidelines recommend reducing the amount of oil consumption, avoidance of deep-fried foods and opting for raw, steamed, or boiled foods instead.^144^^,^^145^ Moreover, mustard oil with its higher alpha-linoic acid, and monounsaturated and polyunsaturated fatty acid content is a recommended substitute for butter, ghee, and vegetable oil. which are high in saturated and trans fats. The use of mustard oil is associated with lower BMI and lower prevalence of ASCVD,^128^^,^^146^ but long-term data are needed to evaluate its beneficial effect. Culturally tailored dietary counselling and motivational strategies have been beneficial in SA diaspora and may help overcome barriers to healthy nutrition.^125^

Aerobics, resistance, and high-intensity interval training have all shown similar effects on reducing hepatic fat independent of weight loss. Interventions aimed at increasing the level of physical activity should consider cultural factors, acculturation level, health literacy, and social networks.^59^ In Asians with T2D, strength training is associated with a superior glycemic control than aerobic training alone.^147^ Culturally preferred physical activity recommendations such as traditional dances and popular sports like cricket and badminton may improve adherence.

Social networks have been shown to influence lifestyle and dietary patterns in adults and adolescents. Family and local community form key constituents of social networks for SA residents and nonresidents alike, influencing access to resources, education, and other socioeconomic constraints. Data from the MASALA study suggests that having an adult child in the social network of older nonresident SAs has a positive influence and facilitates behavioral change in dietary and exercise patterns.^148^ Another cross-sectional analysis of MASALA participants found higher physical activity in SAs who exercised with partners from the social network. For women, the findings were only significant if the exercise partner was a spouse.^149^ Family- and social network–focused strategies may enable sustainable lifestyle interventions that modify ASCVD process and merit further exploration. The efficacy and feasibility of such lifestyle interventions need to be validated for the resident SA populations. Moreover, the heterogeneity within subgroups of SAs makes lifestyle counselling challenging for under-represented communities.

Majority of SAs live in low- and middle-income countries and a large subset resides rurally. Several model-of-care approaches in South Asia have demonstrated significant improvement in cardiovascular risk factors. Opportunistic delivery of intensive cardiovascular screening, structured lifestyle interventions, and active follow-up to spouses and first-degree relatives of patients diagnosed with CAD before 55 years of age is associated with significantly higher odds of optimal control of blood pressure and dyslipidemia as well as abstinence from tobacco at 2 years (OR: 2.2; 95% CI: 1.7-2.7; *P <* 0.0001).^123^ Household-based dietary counselling, home visits, and general awareness sessions are superior to general dietary leaflets in dramatically improving household consumption of salt, sugar, and oil by 45%, 40%, and 48%, respectively.^150^ Community health worker home visits for blood pressure monitoring and counselling, physician training, and care coordination through local public hospitals is associated with greater blood pressure control among rural communities at 24 months.^151^

### Pharmacotherapy

Statins are the first-line LDL-C lowering agents for management of dyslipidemia and in the prevention of ASCVD. They are well-tolerated and equally efficacious among SAs in lowering LDL-C levels.^72^ The LAI and the Sri Lankan National Dyslipidemia Management Guidelines concur with other international guidelines aimed for Caucasians and recommend similar therapeutic LDL-C targets in for various risk groups. The LAI additionally details Apo-B as a secondary treatment target. Despite their propensity toward a maladaptive lipid profile with a “normal” LDL-C, it remains unclear whether SAs benefit from tighter lipid targets, and the utility of alternate biomarkers such as ApoB/ApoA1 ratio for enhanced therapeutic monitoring is also unknown. Moreover, low HDL-C is the most prevalent dyslipidemia among SAs,^19^ and HDL-C particles in this population are thought to be less functional.^72^ Consequently, the efficacy of novel lipid-lowering therapies in the SA context warrants further investigation—not only in raising HDL-C levels, but also in improving HDL functionality, and ultimately reducing long-term ASCVD risk.

The Indian guidelines on hypertension endorse a lower blood pressure target of <130/80 mm Hg for patients under the age of 60 years but recommend initiating pharmacotherapy once blood pressure reaches 140/90 mm Hg.^152^ Among those with known ASCVD or heart failure, the guidelines recommend early initiation of antihypertensive agents at 130/80 mm Hg. It is unclear whether early initiation of pharmacotherapy for moderate- to high-risk SAs without overt hypertension (>140/90 mm Hg) reduces the risk of future ASCVD, and further long-term data are needed to assess this. There have been several instances where certain ethnic groups derive greater therapeutic efficacy from an agent.^153^^,^^154^ Despite the burden of ASCVD, no such ethnic polymorphism exists for SAs, which is in part because of profound under-representation in trials. Across all major cardiovascular randomized control trials published from 2018-2022, SAs made up only 0.47% of the study participants, and none had published SA-specific data or post hoc analyses.^155^ Greater SA recruitment in clinical trials and SA-specific validation studies are warranted to identify ethnicity-specific interventions and optimize therapeutic treatment targets.

Given the higher rates insulin resistance observed, SAs may derive superior therapeutic benefit from certain antidiabetic agents. The South Asian Federation of Endocrine Societies recommend the use of sulfonylureas as the drug of choice in management of diabetes in SAs because of increased efficacy, pleotropic effects, low cost, and safety.^156^ Although other observations suggest that SAs may respond better to incretin-based agents such as dipeptidyl peptidase-4 inhibitors and glucagon-like peptide-1 analogues. The higher rates of beta cell dysfunction, higher postprandial glycemia, high-carbohydrate diet, and onset of T2D at lower BMI further complicates decision-making.^59^ Although some SA-specific management algorithms have been proposed,^157^ head-to-head trials are needed to ascertain optimal therapies. Pharmacogenomic studies may further elucidate the genetic contribution to variability in treatment response and help optimize therapy for SAs with T2D.

## Limitations of Current Literature

Contemporary literature has several limitations that need consideration (Table 3). Most of the literature is based on nonresident SAs in the United States, Canada, and Europe, while studies on resident SAs are scarce and largely confined to India. Participants tend to be middle-to-older aged individuals with high-education and high-income backgrounds, clustered within the metropolitan areas of developed countries. They may exhibit a degree of health literacy, may already engage in preventative care, and are more likely to adhere to interventions. Given that one-quarter of SAs develop ASCVD before the age of 40 years,^8^ the utility of such findings among high these high-risk cohorts and those with lower socioeconomic background needs to be explored further. Moreover, much of the literature may not be generalizable to the overwhelming majority of resident SAs. Despite over 98% of SAs residing natively,^158^ there are no long-term prospective cohort studies in South Asia. Local data are needed to validate these findings observed in the diaspora and better understand the socioenvironmental influences contributing ASCVDs among resident SAs. Significant heterogeneity exists within the SA population depending on the country of origin. Bangladeshi and Pakistani ethnicities confer the greatest risk of ASCVD; yet, their representation among the sentinel study cohorts is limited.^98^ Data focusing on ASCVD among Nepali and Sri Lankan individuals is even scarcer. Local validation of existing literature is also warranted to verify the accuracy of novel risk factors and the efficacy of proposed interventions.

**Table 3:** Main Limitations of the Current Literature Exploring ASCVD in SAs and Future Directions to Address Them

Limitation	Future Directions and Solutions to Address Limitations
Overall paucity of cardiovascular research originating from SA countries	• Establishing academic infrastructure to promote research culture among medical students, junior clinicians, as well as nurses • Advocacy at a public health and policy level to fund grassroots level research • Lobbying the private sector (ie, international pharmaceutical institutes and philanthropic arms of large corporations) to help establish foundations of research in urban and rural SA for robust SA-specific research output • Healthy representation of SA-based clinical and research institutes in large multicenter trials to improve SA participation and produce robust SA-specific outcomes from these trials • Promoting trial leadership within SA • Balanced inclusion of SA leaders in cardiovascular research among international steering committees
Under-representation of SAs in cohort studies	• Inclusion of more SA (especially resident SA) participants in global and regional cardiovascular studies, which requires building clinical trial capability in these regions • Reporting of SA-specific data in post hoc analyses • Development of SA-specific risk prediction models
Over-reliance on retrospective and U.S.-centric data sets	• Establishment of additional prospective SA data sets and registries (preferably from within SA) • Prospective validation studies based in SA countries
Heterogeneity within SA subgroups limits generalizability to all SA communities	• Promoting dedicated subgroup-level research to improve risk assessment and targeted interventions • Nuanced categorization of SAs participating in trials and registries (ie, by country of origin, language, cultural subgroups) • Transitioning away from self-reported ethnicity and toward genomics-based risk stratification in academia • Spotlighting grassroots-level cardiovascular research originating from SA
Limited data on premature-onset ASCVD and understudied unique risk factors	• Longitudinal studies focusing on early-life exposures, psychosocial stressors and lifestyle factors in younger SA cohorts. • Exploring the utility, feasibility, and cost-effectiveness of population-wide screening for preclinical disease (ie, national CAC screening program akin to common solid cancers)
Gaps in genetic and epigenetic drivers	• Greater SA inclusion in genomic studies and development of SA-specific polygenic risk scores to guide precision medicine
Lack of interventions that confer added efficacy among SA populations	• Exploring lifestyle interventions and counselling strategies that can be applied to a linguistically, culturally, and socioeconomically diverse cohort • Tailored clinical trials and SA-centric prospective studies exploring efficacy of key lifestyle and pharmacology interventions • Establishment of region-specific SA guidelines and targets for anthropometric measures beyond BMI and cardiometabolic biomarkers (ie, exploring tighter blood pressure, glycemic control, and lipid targets) • Exploring long-term data into attenuating preclinical disease (ie CAC progression) and its link with the development symptomatic ASCVD • Investigating the interacting effect of novel therapeutics among SAs through clinical trials

ASCVD = atherosclerotic cardiovascular disease; other abbreviations as in Table 2.

Within each country, South Asian communities have diverse subgroups often insulated by regional, cultural, and geopolitical differences, and this poses a key challenge for accurate ASCVD risk assessment and modification. Each subpopulation often demonstrates unique dietary and lifestyle practices, socioeconomic standings, and a contained gene pool. Contemporary literature rarely discriminates between these diverse subgroups that likely exhibit substantial heterogeneity in the prevalence of risk factors, cardiometabolic profile and genetic variants that influence ASCVD risk and outcomes.^69^ Migration within and outside of South Asia further confounds this risk. More sophisticated patient profiling of SA registries and databases are needed to better understand and address the community-specific risk.

Sustained efforts are also warranted to address the aforementioned under-representation of SAs in clinical trials. Asian participation in key cardiovascular, obesity, and diabetes trials of the recent decade is low (8.3% of all trial participants) but has demonstrated a positive correlation with trial collaboration and lead authorship from the associated region (Asia Pacific).^159^ Unfortunately, SA collaboration on major cardiometabolic trials is extremely low (0.8%) and did not increment substantially between 2011 and 2020.^159^ The development of academic infrastructure is necessary to promote trial collaboration and leadership within South Asia and drive SA enrollment. Table 3 details some possible avenues to address these key limitations. Improved SA participation will ultimately enable a reliable evaluation of SA-specific outcomes and interacting effects of therapeutic interventions.

Race and ethnicity are social constructs that underpin one’s socioeconomic standing, environmental influences, and lifestyle practices, which may be different to their inherited genetic risk (ancestry). The downstream effects of social determinants of health may explain much of the racial variation in the prevalence of risk factors and the incidence of ASCVD observed, with education and place of birth being most statistically influential.^148^^,^^160^^,^^161^ Contemporary literature is shifting the focus away from race-based corrections to socioeconomic parameters, which is best reflected in the omission of race and inclusion of zip-code social deprivation index in the new PREVENT equations.^97^ The U.S. Food and Drug Administration still mandates self-reported race and ethnicity data in clinical trials where South Asians are broadly recognized as Non-Hispanic Asians.^162^ Using South Asian race as a risk-enhancing factor in the clinical context may lead to impersonalized management strategies and ethnic profiling that discourages health care engagement.^32^ To improve care and outcomes for SAs, clinicians need to consider the totality of socioeconomic factors in their management approach. The development and implementation of socioeconomically mindful risk assessment approaches for resident SAs remains a linguistic, financial, and geopolitical challenge. Furthermore, as genetic admixture increases in subsequent generations of migrants, self-reported ethnicity becomes a less reliable indicator of one’s genetic risk,^163^ which current assessment tools fail to capture adequately. Incorporating multiethnic PRS in composite risk assessment may improve stratification among SAs.

## Conclusions

SAs remain at a disproportionately high risk of ASCVD, driven by a complex interplay of genetic, metabolic, and environmental factors. Despite significant advances in understanding and quantifying these risks, traditional risk assessment tools do not accurately capture the unique vulnerability of this population. Emerging biomarkers, imaging techniques, and genomics-based approaches hold promise for improving risk stratification and guiding targeted therapies. However, their clinical utility is currently limited and requires further validation in a large, diverse cohort of resident and nonresident SAs.

Future efforts should focus on integrating these findings into personalized primary prevention strategies, emphasizing culturally tailored lifestyle interventions, and addressing systemic barriers to equitable health care. Collaborative, multidisciplinary research remains essential to translate scientific discoveries into meaningful clinical and public health advances in reducing the burden of ASCVD among SAs and other high-risk populations.

## Funding Support and Author Disclosures

The authors have reported that they have no relationships relevant to the contents of this paper to disclose.
